# Myelodysplastic Neoplasms (MDS): The Current and Future Treatment Landscape

**DOI:** 10.3390/curroncol31040148

**Published:** 2024-04-03

**Authors:** Daniel Karel, Claire Valburg, Navitha Woddor, Victor E. Nava, Anita Aggarwal

**Affiliations:** 1 Department of Hematology/Medical Oncology, The George Washington University, Washington, DC 20037, USA; cvalburg@mfa.gwu.edu (C.V.); anita.aggarwal@va.gov (A.A.); 2 Department of Pathology, The George Washington University, Washington, DC 20037, USA; nwoddor@mfa.gwu.edu (N.W.); victor.nava@va.gov (V.E.N.); 3 Department of Pathology, Washington DC VA Medical Center, Washington, DC 20422, USA; 4 Department of Hematology/Medical Oncology, Washington DC VA Medical Center, Washington, DC 20422, USA

**Keywords:** myelodysplastic neoplasm, myelodysplastic syndrome, MDS, precision medicine, targeted therapy

## Abstract

Myelodysplastic neoplasms (MDS) are a heterogenous clonal disorder of hemopoietic stem cells characterized by cytomorphologic dysplasia, ineffective hematopoiesis, peripheral cytopenias and risk of progression to acute myeloid leukemia (AML). Our understanding of this disease has continued to evolve over the last century. More recently, prognostication and treatment have been determined by cytogenetic and molecular data. Specific genetic abnormalities, such as deletion of the long arm of chromosome 5 (del(5q)), TP53 inactivation and SF3B1 mutation, are increasingly associated with disease phenotype and outcome, as reflected in the recently updated fifth edition of the World Health Organization Classification of Hematolymphoid Tumors (WHO5) and the International Consensus Classification 2022 (ICC 2022) classification systems. Treatment of lower-risk MDS is primarily symptom directed to ameliorate cytopenias. Higher-risk disease warrants disease-directed therapy at diagnosis; however, the only possible cure is an allogenic bone marrow transplant. Novel treatments aimed at rational molecular and cellular pathway targets have yielded a number of candidate drugs over recent years; however few new approvals have been granted. With ongoing research, we hope to increasingly offer our MDS patients tailored therapeutic approaches, ultimately decreasing morbidity and mortality.

## 1. Introduction

Myelodysplastic neoplasms (MDS) were first reported in the 1920s by Di Guglielmo, who described patients with cytopenias and abnormal appearing erythrocytes. Over the last 100 years, our understanding of these clinical entities has improved drastically, as reflected in the progression from the primary symptomatic terminology used during the early 20th century to the morphologic definitions used in the French American British (FAB) system and, most recently, to the more complex classification systems incorporating blast count and molecular alterations. Currently, 2 competing MDS classification schemes are accepted: that of the 5th edition of the World Health Organization Classification of Hematolymphoid Tumors (WHO5) and that of the International Consensus Classification 2022 (ICC 2022), which will hopefully be unified as our understanding of the biology of MDS continues to improve. The evolution of treatment, from improvements in supportive care to the development of newer therapies targeting molecular derangements, has reflected our increasing understanding of the pathogenesis of this heterogenous disease. As the field moves forward we hope that both supportive as well as disease directed therapies become increasingly tailored to patient specific characteristics.

## 2. Molecular Evolution of MDS from Genotype to Phenotype and Prognostication

Our understanding of the biology of MDS remains incomplete and is constantly refined based on research correlating genetic alterations with clinical phenotypes, as reflected in the current WHO5 and ICC 2022 classifications. It is well established that the development of MDS involves the multistep acquisition of genomic alterations arising in hematopoietic stem cells. The most common mutations occur in genes involved in RNA splicing (SF3B1, SRSF2, U2AF1 and ZRSR2), epigenetic modification (TET2, ASXL1 and DNMT3A), signal transduction regulation (NRAS and JAK2) and transcription (RUNX1 and TP53) [1]. Some of the more common mutations seen in MDS are listed in Table 1 [2,3,4,5,6,7].

Although recognizable MDS mutations have been described in more than 40 genes, reliable genotype-to-phenotype relationships are identified in less than 5 percent of cases. For example, mutations in SF3B1 are associated with MDS with ringed sideroblasts (MDS-RS), and mutations in SRSF2 are associated with CMML. As another example, Seethy et al. in 2023 demonstrated a reduction in TET2 expression among patients with high- or very-high-risk MDS per the Revised International Prognostic Scoring System (IPSS-R) and myelodysplasia-related AML (AML-MR in WHO5), as compared to patients with low- or intermediate-risk. The authors also noted that levels of 5-hydroxymethyl cytosine were concordantly lower [8]. The ICC 2022 recognizes MDS founding lesions and mutations in the following genes: ASXL1, BCOR, EZH2, RUNX1, SF3B1, SRSF2, STAG2, U2AF1 and ZRSR2 [9].

Karyotypic abnormalities correlate in some cases with specific phenotypic features that influence management decisions and modify outcomes. It is well known that MDS with isolated deletion of the long arm of chromosome 5 (del(5q)), with or without additional cytogenetic aberrancy (except monosomy 7) responds well to lenalidomide, portending a superior prognosis [10]. Conversely, certain chromosomal abnormalities, such as deletion of 17p, associated with the loss of TP53, independently predict poor prognosis and resistance to treatment. Other common chromosomal aberrations in MDS include del(7q), trisomy 8, del(20q) and complex karyotype (CK). CK, which is also frequently associated with TP53 pathogenic alterations, has yet to be uniformly defined. The definition of CK can vary geographically and nosologically, although in most studies CK is generally defined as the presence of ≥3 independent cytogenetic abnormalities. However, the Medical Research Council Acute Myeloid Leukemia 10 trial (MRC AML10) required the presence of ≥5 independent cytogenetic abnormalities. It is known that CK is associated with a poor prognosis in hematological malignancies [11].

Research into the molecular pathology of MDS has exploded recently, spearheaded in part by advances in massively parallel sequencing techniques. In 2011, a seminal study by Bejar et al. examined DNA from 439 cases and identified point mutations in 18 genes. They discovered that TP53 mutations were observed mainly in patients with intermediate-2 or high-risk disease according to the International Prognostic Scoring System (IPSS) and described a correlation with thrombocytopenia, an increased number of blasts and CK. Furthermore, severe thrombocytopenia and an increased number of blasts were more prevalent when mutations in RUNX1, TP53 and NRAS were present. Combined alterations in 5 or more genes were associated with an increased risk of death from any cause, and the specific hazard ratio for specific genetic alterations was reported as follows: TP53 (2.48), EZH2 (2.13), ETV6 (2.04), RUNX1 (1.41) and ASXL1 (1.38) [12].

More recently, Bersanelli et al. identified distinct MDS clusters linked to specific genomic features, defining distinct genomic groups with unique outcomes: no specific genomic abnormalities, SF3B1 related, TP53/complex karyotype, SRSF2 related, U2AF1 associated 20q deletion or chromosome 7 abnormalities, and finally AML-like mutation patterns. Isolated SF3B1-related MDS portend superior survival [13]. The authors also noted that specific co-mutation patterns accounted for clinical heterogeneity within SF3B1- and SRSF2-related MDS. Unsurprisingly, MDS with CK and/or TP53 gene abnormalities and MDS with AML–like mutations were associated with inferior prognosis. Interestingly, MDS with del(5q) were clustered into 2 distinct subgroups according to the number of mutated genes and/or presence of TP53 mutations. The study concluded that individuals with del(5q) who have either no additional mutations or only a single additional mutation tend to have a more favorable prognosis compared to that of those with coexisting TP53 mutation [13].

## 3. Prognostication

MDS prognostication based on genomic information is evolving. The IPSS-R is widely used and describes 5 categories ranging from very-low- to very-high-risk, which uses cytogenetic as well as hematologic parameters, including bone marrow blast count, hemoglobin levels, platelet count and absolute neutrophil count [14].

In 2022, the Molecular IPSS for Myelodysplastic Syndromes (IPSS-M) was proposed as a risk stratification tool based on the combined profiling of 152 genes along with clinical data, representing a major advancement in the field. We consider the IPSS-M to be the heir to the IPSS-R. The IPSS-M utilizes 6 prognostic categories and re-stratified nearly half (46 percent) of 1281 cases of MDS, including not only primary but also secondary and therapy-related cases, demonstrating improved risk stratification and prognostic accuracy based on a refined list of 31 mutated genes [15,16]. The IPSS-M has also been noted to be a more accurate predictor of outcomes (leukemia-free survival and overall survival) compared to the IPSS-R [17]. Notably, the IPSS-M allows for missing data facilitating its practical application in settings in which routine genomic testing is still unavailable.

## 4. Classification 

The revised WHO5 and ICC 2022 both reflect our increased understanding of the impact of genomics on the clinical presentation of MDS. For example, in the ICC 2022 classification, MDS-RS has been replaced by an entity called MDS with mutated *SF3B1.* Likewise, the new schema differentiates MDS with mutated TP53 (0–9 percent blasts) from MDS/AML (with mutated p53 and 10–19 percent blasts), because the presence of multi-hit *TP53* mutations in cytopenic myeloid neoplasms corresponds to a highly aggressive disease with a short survival period, regardless of morphologic features including blast count [12]. Table 2 lists the recognized MDS subgroups according to both the WHO5 and ICC 2022 [9,18].

## 5. Treatment of Lower-Risk Disease

MDS are clinically diverse entities. They can present as incidentally discovered mild anemia or as symptomatic disease in the setting of bone marrow failure. Symptoms classically include fatigue, infections and bleeding. The goals of therapy for lower-risk MDS are generally aimed at reducing symptoms, improving patients’ quality of life, decreasing the transfusion burden and preventing other complications, such as iron overload, infections or bleeding. Observation is universally accepted as the management modality of choice in asymptomatic patients with low-risk disease.

The following discussion will review the evidence supporting treatment modalities for “lower-risk” MDS, which encompass patients classified as very low-risk, low-risk and intermediate-risk (with 3.5 points or less), according to the IPSS-R, as suggested by Pfeilstöcker et al. [19], and as incorporated into the NCCN guidelines [20].

Although the correlation is not perfect, the WHO5 categories including MDS with low blasts with an SF3B1 mutation and MDS with low blasts with an isolated del(5q) (as well as the corresponding categories in the ICC 2022 classification), generally correspond to the IPSS-R very-low- or low-risk categories. The WHO5 entity MDS-h (hypoplastic) generally falls into the IPSS low-risk category as well [21].

### 5.1. Blood Product Transfusion

An international survey of patients conducted by the MDS Foundation revealed that approximately 75 percent of all patients with MDS in Europe and the U.S. receive packed red blood cell (pRBC) transfusions [22]. Patients are generally transfused in order to minimize symptoms and keep hemoglobin (Hb) levels within a safe, near physiologic range. Often a Hb level of 7.0 or 8.0 gm/dL is used as a trigger for pRBC transfusion based on expert opinion, although prospective clinical data are lacking. The absence of standardization for pRBC transfusion impedes a thorough cost–benefit analysis of this approach, which represents a high financial expense for the healthcare system [23].

Transfusion dependence in MDS is associated with lower leukemia-free survival and overall survival rates, irrespective of the calculated risk score, and is (unsurprisingly) associated with a subjective lower quality of life [23]. Transfusion-dependent patients are at risk of complications, most notably, transfusion-associated circulatory overload (TACO) and transfusion-related acute lung injury (TRALI). Furthermore, there is a significant risk of developing iron overload, a notable chronic complication of pRBC transfusions, although this may be reduced with chelation therapy. Currently, deferoxamine, deferiprone and deferasirox are both FDA- and EMA-approved iron chelators. The NCCN recommends considering deferoxamine or deferasirox for patients who have received greater than 20 to 30 units of pRBC, and to decrease ferritin levels to less than 1000 ng/mL for patients with serum ferritin levels greater than 2500 ng/mL [20].

The TELESTO trial, a phase II study published by Angelucci et al. in 2020, included participants with lower-risk MDS who received between 15 and 75 pRBC transfusions with elevated ferritin levels. Iron chelation with oral deferasirox resulted in an improved event-free survival (EFS) rate, although the study was not powered to show survival benefit [24]. Given the high proportion of patients who receive transfusions, determining who will benefit most from chelation therapy is an important goal.

Although up to 65 percent of patients with MDS have thrombocytopenia, routine platelet transfusions outside of certain clinical situations (such as bleeding or peri-procedural) are not currently recommended in the treatment guidelines. There are no clinical trials that we are aware of exploring outcomes of platelet transfusion in patients with low-risk MDS and clinical triggers can vary widely in practice.

### 5.2. Erythropoiesis Stimulating Agents (ESA)

Erythropoietin (EPO), an ESA, is an established treatment modality for anemia in patients with lower-risk MDS [23]. EPO alone is expected to improve anemia in 15 to 20 percent of allcomers with MDS, as shown by Hellstrom-Lindberg et al. in a 1995 meta-analysis that included 205 participants [25]. However, the authors noted that certain participants seemed to derive more benefit from EPO therapy than others. For example, those without a previous transfusion requirement and those with lower endogenous EPO levels (below 200 mU/mL) were more likely to respond to EPO therapy. The authors noted a lack of response to EPO therapy in participants with refractory anemia with ringed sideroblasts (RAS) and with an EPO level above 200 mU/mL. Participants with RAS and with EPO levels below 200 mU/mL who did not previously require transfusion showed response rates of about 33 percent. In contrast, participants without RAS and without previous transfusion requirement showed response rates above 50 percent. These findings nicely demonstrate the utility of using readily available clinical data in order to determine the potential efficacy of a treatment modality.

Hellstrom et al. demonstrated that the combination of EPO and granulocyte colony-stimulating factor (G-CSF) improved hemoglobin levels in approximately 38 percent of participants with MDS, indicating a synergistic effect between these therapies [26]. Notably, those with RAS had the highest response rate (46 percent) to combination therapy and those with refractory anemia (RA) had the lowest (20 precent). When using a validated decision model including the pretreatment pRBC transfusion burden as well as the EPO level, a certain subgroup (with an EPO level below 500 mU/mL and receiving less than 2 units pRBC per month) responded to the combination particularly well, with a 61 percent response rate [27].

Investigators more recently have continued to explore ways to improve anemia in MDS patients using ESA therapy. Darbepoetin is a longer-acting ESA, with a half-life approximately 3 times that of EPO. Platzbecker et al. published a randomized-placebo controlled trial in 2017 evaluating the use of darbepoetin in 147 participants with anemia and lower-risk MDS and found that the pRBC transfusion requirement was significantly lower with darbepoetin compared to placebo (36.1 percent vs. 59.2 percent) [28]. No new safety concerns were raised, and the study included a 48-week open-label extension study in which most of the participants received darbepoetin every 2 weeks (as opposed to every 3 weeks) and demonstrated improved hematologic response.

Providing more recent evidence, Fenaux et al. in 2018 published a randomized, controlled trial assessing the efficacy of ESA therapy in 130 participants with lower-risk MDS. Participants were randomized in a 2:1 fashion to receive ESA (epoetin-alpha, 450 IU/kg/week) or placebo for 24 weeks, followed by a treatment extension period in the responders. The primary endpoint was erythroid response (using the modified International Working Group (IWG)-2006 criteria) at week 24, and a response rate of 45.9 percent in the treatment group vs. 4.4 percent in the placebo group was observed [29]. The ESA-treated group had a reduced RBC transfusion requirement and increased time-to-first-transfusion compared to the placebo group.

Notably, the European Medicines Agency (EMA) has not approved darbepoetin in this setting, despite its approval by the FDA in the United States, and there are currently no data comparing different ESA formulations head-to-head.

### 5.3. Thrombopoietin Receptor Agonists (TPO-RA)

Thrombocytopenia poses a significant clinical problem in patients with lower-risk MDS. It has been estimated that the prevalence of thrombocytopenia is between 40 and 65 percent. Thrombocytopenia is an adverse risk factor associated with an increased risk of bleeding, and the risk of thrombocytopenia increases with IPSS category, with the highest prevalence of thrombocytopenia seen in the high-risk category [30]. Platelet transfusion has limited therapeutic utility and is associated with transfusion-related adverse effects. The role of TPO-RA therapy in MDS has yet to be fully elucidated, and studies have demonstrated variable benefit. TPO-RAs such as romiplostim and eltrombopag have been shown to facilitate megakaryocyte differentiation and increase platelet count in diseases such as immune thrombocytopenia (ITP), aplastic anemia and certain hematological malignancies, such as CML. At the present time, the use of TPO-RA therapy for MDS specifically remains under investigation, although there are studies that suggest benefits.

The efficacy and safety of eltrombopag was explored in a phase II trial published in 2017 by Oliva et al., in which platelet responses (with a median follow-up time of 11 weeks) occurred in 47 percent of participants who received eltrombopag versus only 3 percent in the placebo group. The authors concluded that this drug was well tolerated and clinically effective [31]. During the initial follow-up period, 21 trial participants experienced a severe bleeding event (as defined by a WHO bleeding score of 2 or more), 13 of whom were enrolled in the placebo arm, representing a statistically significant difference between the groups.

A follow-up to this trial published in 2023 confirmed the longer-term efficacy and safety of eltrombopag. The results at week 11 and week 25 were indistinguishable, and the updated data showed that 25 percent of participants who responded to eltrombopag at week 25 stopped responding by 60 months [32]. However, a statistically significant decrease in bleeding events was maintained in the treatment arm.

Providing further evidence for eltrombopag, a phase II dose-escalation study (starting at 50 mg daily and increasing to a maximum dose of 150 mg over 16 weeks) in participants with lower-risk MDS showed a hematologic response between 16 and 20 weeks in 11 of the 25 participants, as per the IWG definition. The predictors of response were the presence of a paroxysmal nocturnal hemoglobinuria (PNH) clone, marrow hypocellularity, thrombocytopenia and elevated plasma thrombopoietin levels at study entry [33].

Similarly, romiplostim can improve platelet counts by approximately 40 to 50 percent, reduce platelet transfusions and decrease bleeding risk in MDS patients with lower-risk disease [34]. However, the long-term safety and efficacy of this drug are not fully elucidated. An open-label extension study by Fenaux et al. published in 2017 included 60 participants from previous TPO-RA trials [35,36,37] with lower-risk MDS and a platelet count of 50,000 or less. They used romiplostim for a mean duration of 57 weeks. Treatment-related adverse effects (AEs) were found in 30 percent of the participants, and progression to AML occurred in 2 participants after 44 and 46 weeks. The median response duration was 33 weeks, and 82 percent of participants had a continuous response without new safety concerns, demonstrating longer-term safety [34].

In 2020, a systematic review and meta-analysis from Meng et al. analyzed 8 studies with over 1000 participants collectively, including lower-risk and higher-risk MDS (and AML), treated either with romiplostim or eltrombopag. The authors found that TPO-RAs decreased all bleeding events (including grade 3 or 4 events) without increasing the rate of AML transformation [38]. However, a decrease in the overall response rate (ORR) in participants receiving TPO-RA therapy was detected, suggesting that TPO-RAs may have a detrimental effect on hematologic indices, seemingly more pronounced with eltrombopag and in those with higher-risk disease. Different treatment regimens used in dissimilar studies (e.g., azacitidine vs. decitabine) may have contributed to this finding. Overall, more data are needed to determine if this therapy should be routinely incorporated into treatment paradigms and to determine which subset of MDS patients is most likely to benefit from therapy.

### 5.4. Luspatercept

Luspatercept is a recombinant fusion protein that binds to certain transforming growth factor β (TGF-β) superfamily ligands, decreasing SMAD protein signaling and ultimately accelerating erythroblast maturation. Although luspatercept was approved by the FDA in 2019 for beta-thalassemia, its use has been extended to include treatment of anemia in patients with MDS-RS and in lower-risk MDS in 2020 and 2023, respectively. Luspatercept was approved by the EMA in June 2020.

The 2017 PACE-MDS open-label, phase II trial by Platzbecker et al. included participants with lower-risk MDS with and without increased RS. High rates of hematologic response and decreased transfusion requirements were observed in luspatercept-treated participants with lower-risk MDS, which was notably more striking in those with MDS-RS and MDS with SF3B1 mutation (hematologic response rates of 69 percent and 77 percent, respectively) [39].

In November of 2022, an update to the PACE-MDS trial confirmed the benefit of luspatercept for patients with lower-risk MDS, with an erythroid hematologic response seen in approximately 54 percent of participants per the IWG criteria, without new safety concerns. Specifically, responses were noted in approximately 68, 36 and 71 percent of participants with MDS-RS, MDS without RS and non-transfusion-dependent MDS, respectively [40].

The phase III, placebo-controlled MEDALIST trial included participants with lower-risk MDS who had been receiving frequent transfusions and who were less likely to respond to ESA therapy. 38 percent of those who received luspatercept were transfusion-independent for 8 weeks or longer [41].

The interim results of the highly anticipated open-label, phase III randomized COMMANDS trial were recently published in July 2023 and included transfusion-dependent, ESA-naïve participants with lower-risk MDS (regardless of RS subtype). Participants received either epoetin alfa or luspatercept, and at 24 weeks of treatment, 59 percent of those in the luspatercept arm had reached the primary end point (an increase in Hb of 1.5 g/dL and transfusion-independence for at least 12 weeks), in comparison to only 31 percent of those who received epoetin [42]. Although longer-term studies are warranted, this does provide further compelling evidence for the role of luspatercept in the lower-risk setting.

### 5.5. Immunomodulation/Immune Suppression

Immunosuppression therapy is a mainstay of treatment in patients with lower-risk MDS and generally includes lenalidomide and anti-thymocyte globulin (ATG) paired with various agents, including cyclosporine, prednisone and tacrolimus.

Lenalidomide is an immunomodulatory agent with multiple mechanisms of action and is effective in the presence of del(5q) [20]. Fenaux et al. demonstrated the efficacy of lenalidomide in a randomized, placebo-controlled trial in 2011, which included 205 participants with del(5q) and lower-risk MDS. Lenalidomide was shown to significantly increase transfusion independence (TI) at 26 weeks and beyond in a dose-dependent manner (56 percent, 42.6 percent and 5.9 percent TI with 10 mg, 5 mg or 0 mg, respectively) [43].

List et al. examined long-term outcomes from the single-arm MDS-003 study [44] and demonstrated that the rate of TI in lower-risk participants with del(5q) 8 weeks after receiving lenalidomide was 65.5 percent, with 63 of 88 evaluable participants achieving a partial or complete cytogenetic response. Importantly, the duration of response was sustained, with a median duration of response of 2.2 years. TI was associated with an overall survival benefit and increased time to the development of AML [45].

In 2016, Santini et al. demonstrated that lower-risk patients without del(5q) may benefit from the use of lenalidomide as well. The authors noted a 26.9 percent response rate in achieving RBC-TI in this population, as well as an improved quality of life [46]. Of note, this study included participants who were transfusion-dependent and refractory or otherwise not eligible for ESA therapy.

Immune suppression, including ATG and cyclosporine, is currently incorporated in the NCCN guidelines in lower-risk patients with elevated endogenous EPO whose cytopenia would be expected to improve with immunosuppressive therapy [20]. Although it has long since been proposed that T-cell-driven immunity may play a role in MDS-associated cytopenias, large trials to prove these therapies beneficial are still lacking.

A retrospective analysis published by Stahl et al. in 2018 explored the outcomes of immune suppression therapy from multiple centers in the U.S. and Europe. The authors found that the most commonly utilized regimen was ATG with prednisone (43 percent), followed by ATG plus cyclosporine (21 percent). ATG was also paired with tacrolimus and etanercept, among other agents. In general, the overall response rate to immunosuppressive therapy (three-quarters of participants were treated with ATG) was approximately 49 percent, and approximately 11 percent achieved a complete response, while 30 percent achieved transfusion independence [47]. The presence of hypocellular marrow was associated with transfusion independence.

A 2014 non-randomized, phase II trial by Komrokji et al. demonstrated that rabbit ATG resulted in one-third of the participants achieving a durable hematologic response following 4 daily doses of therapy. Interestingly, participants with a higher percentage of CD8 memory T-cells, as well as CD4 T-cells with a higher proliferative index, were more likely to benefit from treatment with ATG [48].

### 5.6. Hypomethylating Agents (HMA)

Hypomethylating agents (HMAs) are currently used in AML for transplant-ineligible patients who are not candidates for intensive chemotherapy. HMA therapy is also well established as frontline treatment in patients with high-risk MDS [20], as discussed in subsequent sections. However, there are data to suggest that it may be beneficial in lower-risk disease as well. The use of HMA therapy is currently included in the NCCN guidelines for patients with lower-risk MDS without del(5q) for symptomatic anemia with elevated endogenous EPO levels (>500) and a low likelihood of hematologic response to immunosuppressive therapy [20]. Despite this, HMA therapy is not approved for use in Europe in the lower-risk setting.

Several trials over recent decades have demonstrated efficacy and safety using lower doses of HMA in lower-risk MDS. In 2017, Jabbour et al. compared 20 mg/m^2^ decitabine to 75 mg/m^2^ azacitidine, with both drugs given daily for 3 consecutive days. Although the authors concluded that using lower doses of HMA were safe and effective in general, there was a significantly increased ORR and a significantly higher cytogenetic response rate with decitabine compared to azacitidine, especially among participants with higher-risk features, such as mutations in TP53 or ZRSR2. The authors also noted a higher transfusion independence rate with decitabine [49]. A 2020 phase III, placebo-controlled study by Garcia-Manero et al. evaluated the use of oral azacitidine in patients with lower-risk, transfusion-dependent MDS. This study included 216 participants and demonstrated an improvement in the rate of transfusion independence; however, this benefit came at the cost of an increased number of deaths related to infection [50]. Although the use of HMA therapy is accepted in this setting, many questions remain and lower-risk patients should be selected carefully. Furthermore, it is unclear what subsequent therapy should be selected upon HMA failure or intolerance.

### 5.7. Telomerase Inhibition

Telomere length appears to be shorter in mononuclear cells in MDS patients, and perhaps somewhat paradoxically, telomerase activity and expression of human telomerase reverse transcriptase are often increased in MDS cells [51]. It has been hypothesized that this activity may contribute to the growth of the malignant clone, suggesting that telomerase may be an effective therapeutic target.

The efficacy of telomerase inhibition was demonstrated by a single-arm, phase II study of 57 transfusion-dependent participants with lower-risk MDS who relapsed or were refractory to ESA therapy. 37 percent of those treated with the telomerase inhibitor imetelstat achieved transfusion independence at 8 weeks, and 23 percent were transfusion-independent at 24 weeks [52]. The median duration of transfusion independence was 65 weeks. The results appeared to be more robust for participants without del(5q), as well as those who were HMA- or lenalidomide-naïve. Cytopenias, especially thrombocytopenia and neutropenia, were common AEs and occurred in 61 percent and 67 percent of the study participants, respectively.

The phase III iMERGE trial, recently published in January of 2024, explored the efficacy of imetelstat in ESA-relapsed, -refractory or -ineligible participants with lower-risk MDS. This was a multinational, placebo-controlled, double-blind trial that included 178 participants, and the primary outcome was transfusion independence at 8 weeks, which was reached in 40 percent of the treatment arm and 15 percent of the placebo arm [53]. Despite its efficacy in regard to transfusion independence, grade 3 or grade 4 thrombocytopenia or neutropenia occurred in 62 and 68 percent of the participants receiving imetelstat, respectively. This demonstrates the potential role for this agent as a second-line therapy following the administration of ESA. With the recent approval of luspatercept in this space, the optimal sequencing of therapy has yet to be determined.

## 6. Higher-Risk Disease

Patients with higher-risk MDS have IPSS-R intermediate- (with a score of greater than 3.5), high- or very-high-risk disease [19]. Compared to lower-risk MDS, higher-risk MDS carries a worse prognosis with a shorter time to leukemic transformation and a shorter overall survival. Unlike in lower-risk MDS, treatment is typically indicated at the time of diagnosis of higher-risk disease, as outlined in 2013 by Sekeres and Cutler [54]. The WHO5 category MDS with increased blasts (MDS-IB, including MDS-IB1, MDS-IB2 and MDS-f) as well as the ICC 2022 categories MDS with excess blasts (MDS-EB) and MDS/AML (with blast count higher than 10 percent) are likely to be classified as high- or very-high-risk according to IPSS-R. The ICC 2022 category MDS-NOS without dysplasia would also likely fall into the IPSS-R higher-risk category, due to the presence of monosomy 7 or a complex karyotype.

### 6.1. TPO-RA in Higher-Risk Disease

TPO-RA therapy has previously been suggested to be safe and efficacious in the treatment of thrombocytopenia in patients with higher-risk MDS. Platzbecker et al. in a 2015 international, multicenter study demonstrated that eltrombopag had an acceptable safety profile in participants with MDS and AML with no significant increase in blasts within 3 months of treatment initiation [55], and the phase II ASPIRE trial demonstrated that the average number of weekly thrombocytopenic events (defined as the need for platelet transfusion, a grade 3 or higher bleeding event or a platelet count less than 10,000) was significantly lower in those treated with eltrombopag. It is worth noting that 2 fatalities attributable due to the drug were reported [56].

Conversely, the phase III, placebo-controlled SUPPORT Trial published by Dickinson et al. in 2018 showed that the addition of eltrombopag to azacitidine did not improve overall or progression-free survival in higher-risk MDS. Furthermore, participants who received eltrombopag had inferior outcomes, both in terms of survival and platelet transfusion independence [57]. Concerningly, there was also a trend toward progression to AML with eltrombopag therapy. Therefore, we do not find the data for TPO mimetics for higher-risk disease particularly compelling, and there remains no clear role for routine use of these agents at this time.

### 6.2. HMA

Aberrant DNA methylation is a well-described molecular feature of higher-risk MDS. In the NCCN guidelines, HMA therapies, including the DNA methyltransferase inhibitors (DNMTis) azacitidine and decitabine, are first-line agents for patients with higher-risk MDS who are unfit for hematopoietic stem cell transplantation [20]. Azacitidine was initially approved by the FDA for the treatment of MDS in 2004 and by the EMA in 2008. Decitabine became FDA-approved in 2006; however, decitabine remains unapproved for the treatment of MDS by the EMA, as it has not been shown to prolong OS.

A randomized, phase III, multicenter, open-label trial by Fenaux et al. in 2009 assessed the efficacy of azacitidine compared to conventional treatment. The conventional treatment arm in the study included either supportive care, cytarabine or conventional chemotherapy, which was decided prior to randomization based on what was deemed appropriate for each individual. There was an improvement in overall survival in participants who received azacitidine when compared to the “conventional care” arm with a median survival of 24.5 months vs. 15 months, respectively [58]. At the 2-year follow-up, approximately 51 percent of those randomized to the azacitidine arm were still alive, compared to 26 percent of those randomized to the “conventional treatment” arm.

In 2011, a randomized, phase III trial compared decitabine to best supportive care in higher-risk, elderly participants (median age of 70) who were deemed ineligible for chemotherapy. The progression-free survival period was significantly longer in those that received decitabine (approximately 6.5 months) compared to those that received supportive care (3 months). AML transformation at 1 year was also significantly reduced with the decitabine treatment. Importantly, the decitabine treatment was also associated with improved patient-reported quality-of-life metrics. There was no statistical difference in overall survival or acute myeloid leukemia (AML)-free survival (AMLFS) with decitabine treatment [59].

Oral HMAs can potentially decrease the need for transfusions, thereby alleviating the burden of extra-domiciliary treatment. However, developing agents with adequate oral bioavailability has been difficult. A phase II study including 80 participants assessed the bioavailability of oral cedazuridine with decitabine and found that oral cedazuridine with decitabine allowed for similar levels of systemic decitabine exposure as compared with that of a standard dose of IV decitabine. It is important to note that this study only determined the area under the curve (AUC) in the blood and did not assess specific outcomes with this regimen [60]. More data are clearly needed to ensure the efficacy of oral HMA therapy in patients with higher-risk MDS and to determine those most likely to benefit.

### 6.3. Venetoclax

Although HMAs are considered first-line therapy for patients with higher-risk MDS, only about 50 percent of patients respond to these agents. Furthermore, patients who do not respond can develop drug resistance after which prognosis is poor. Venetoclax, an oral selective inhibitor of the anti-apoptotic protein BCL-2, which is utilized in the treatment of AML, has been explored as an additional treatment option for patients with higher-risk MDS.

A retrospective study of 44 patients assessed the impact of venetoclax added to HMA therapy. An overall response to venetoclax was seen in 59 percent of the participants. Encouragingly, a response rate of 44 percent was observed even among patients with previous HMA failure [61]. With this encouraging, albeit limited data, we eagerly await the result of the ongoing phase III, placebo-controlled VERONA trial [NCT04401748] comparing the combination of venetoclax and azacitidine to azacitidine alone in previously untreated participants with higher-risk MDS. The study has completed accrual with 531 participants with an estimated primary completion date of February 2025.

Venetoclax in combination with chemotherapy may also be a therapeutic option in higher-risk MDS patients. Kadia et al. published a phase II study in 2021 investigating treatment with cladribine, idarubicin and cytarabine in combination with venetoclax. This study primarily included participants with AML, although of the 50 study participants, 5 had high- or very-high-risk MDS as per IPSS-R. A total of 47 of the 50 participants achieved either a complete response (CR) or CR with incomplete count recovery (CRi), as defined by the IWG criteria. A total of 37 participants achieved minimal residual disease negativity. Notably, 29 of the 47 responders were eventually taken to transplant. Neutropenic fever was a common grade 3 AE, affecting 42 participants [62].

### 6.4. Chemotherapy

Chemotherapy may be a treatment option in patients with higher-risk MDS. In the NCCN guidelines, chemotherapy regimens similar to those used in AML (such as an anthracycline in combination with cytarabine) are considered, especially before pursuing allogeneic transplant [20]. The role of chemotherapy in MDS treatment is not clear; however, it has been demonstrated that chemotherapy does not benefit patients with a high-risk karyotype, as demonstrated by Knipp et al. in 2007. Participants aged 60 years old or greater with MDS or AML who received intensive chemotherapy were included in this single-center study. Participants with a high-risk karyotype (defined by abnormalities in 3 or more chromosomes or the involvement of chromosome 7) demonstrated a significantly decreased response to intensive chemotherapy. They also had a decreased overall survival (median of 4 months) compared to those with a normal karyotype (18 months). Participants with an abnormal karyotype (but not meeting the criteria for a high-risk karyotype) had an OS of 6 months [63].

To date, comparative studies have not shown the superiority of a particular intensive chemotherapy regimen (including idarubicin-, cytarabine-, fludarabine- and topotecan-based regimens) for patients with MDS [64]. At the present time, the NCCN guidelines suggest chemotherapy is preferred in the setting of a clinical trial, given the lack of proven benefit and the toxicity profile of chemotherapeutic agents.

### 6.5. Allogenic Hematopoietic Stem Cell Transplant (Allo HSCT)

Allo HSCT remains the only potentially curative treatment option for MDS and is the recommended standard of care for any suitable higher-risk candidate. The literature on stem cell transplantation for MDS has recently been reviewed [65]. Some important unanswered questions remain, including determining the optimal pre-transplant conditioning regimen, determining if there is a need for cytoreductive therapy prior to transplant and determining what the optimal cytoreduction treatment would be.

The optimal pre-transplant conditioning regimen in MDS remains to be determined, with relatively few clinical data to guide this decision. While reduced intensity conditioning (RIC) regimens have the benefit of a lower rate of treatment-related mortality (TRM) compared to myeloablative conditioning (MAC) regimens, RIC is associated with a greater risk of disease relapse. Scott et al. demonstrated this in a randomized trial including participants with MDS and AML (less than 5 percent blasts at transplant and with an HLA-matched donor) eligible for either MAC or RIC regimens. The results at the long-term follow-up demonstrated a survival advantage for those who received MAC, which was driven by a decreased relapse rate despite the higher TRM [66]. For patients who cannot tolerate MAC prior to transplant, RIC remains an acceptable option. In 2013, Koreth et al. demonstrated that among MDS patients aged 60–70 years and classified as intermediate-2 or high-risk as per IPSS, RIC transplantation demonstrated an overall and quality-adjusted survival benefit compared to non-transplant therapy [67]. This study also nicely demonstrates that among low- and intermediate-1-risk participants per IPSS, transplantation was associated with markedly reduced survival.

Another unanswered question is whether cytoreduction is necessary prior to proceeding with transplantation. The NCCN currently recommends optimal debulking (with a blast percentage less than 5) prior to allo HSCT, which is especially important if using RIC regimens [68]. There are data that suggest that cytoreduction prior to transplant decreases the rate of relapse; however, there is some controversy in this space as there are also data suggesting that cytoreduction is not necessary to achieve desirable outcomes. For example, Chen et al. compared upfront transplantation to cytoreductive therapy prior to transplantation (including treatment with HMA and treatment with chemotherapy) in participants with MDS-EB-1 and EB-2. Critically, the dropout rate was significantly lower for those treated with upfront transplantation compared to those who received pre-transplant induction chemotherapy or HMA treatment. This study also demonstrated that pre-transplant cytoreductive therapy was correlated with increased TRM and decreased OS [69]. Therefore, upfront transplantation may be preferable for our MDS patients. A retrospective analysis by Schroeder et al. published in 2019 also suggests that upfront transplantation may be preferable to cytoreduction. Among 126 participants with MDS-EB, the 5-year OS was higher in those transplanted upfront compared to those who received chemotherapy or HMA prior to transplantation, although the difference was not statistically significant. Furthermore, the relapse rates between the groups were similar [70]. The authors note that adverse cytogenetics, an RIC regimen and an unrelated donor predicted inferior outcomes, whereas blast count did not influence OS or relapse rates. The authors also note there was a higher 2-year OS rate after relapse in the upfront transplant group. Pre-transplant therapy may select for resistant clones, as those in the upfront transplant group had a higher likelihood of responding to HMA therapy as salvage therapy following a relapse. This suggests that an upfront transplant strategy may be preferrable, given the risk of delaying this curative procedure, and the risk of rendering a patient no longer eligible for transplant due to treatment-associated morbidity.

The role of HMA therapy prior to transplant in MDS patients has not yet been fully elucidated. A meta-analysis published in 2019 by Qin et al. included 6 retrospective cohort studies with a total of 635 patients having undergone transplant, of which 278 received HMA therapy prior to transplantation. The use of HMA before allo HSCT did not improve OS or relapse-free survival (RFS) compared to the control group (which included both chemotherapy as well as best supportive care). However, older patients had significantly increased OS with HMA treatment [71]. Kim et al. published a retrospective analysis in 2014 demonstrating that the OS and RFS were not significantly different between patients who were bridged to transplantation with HMA and those who were not. The non-relapse mortality was also not significantly different between the groups, although it is worth noting that the median age of the HMA group was significantly higher (47 years old) as compared to those who did not receive HMA (41 years old), although both groups had a median age markedly below the average age of MDS diagnosis. Among patients with a blast count above 5 percent at the time of diagnosis, pre-transplant HMA therapy did have a significant non-relapse mortality benefit [72]. In 2019, Modi et al. retrospectively analyzed 172 MDS patients who underwent transplantation. There was no survival benefit seen with the administration of pre-transplant HMA. It is notable that a higher relapse rate was observed in the patients treated with HMA but who did not respond to treatment, likely due to resistant disease [73]. In light of the available evidence, the administration of HMA prior to transplantation should not be routinely considered in patients with higher-risk MDS.

### 6.6. IDH Inhibition

Approximately 5 percent of patients with MDS harbor mutations in isocitrate dehydrogenase (IDH)-1 or -2, with IDH-2 alterations occurring more commonly (the converse is true in AML). In addition, there is mutational stability over time, although new IDH mutations have been reported during leukemic transformation [74]. IDH mutations are associated with older age, higher platelet count and concurrent alteration in DNMT3A, ASXL1 and SRSF2 [75]. Interestingly, mutated IDH-2 is associated with an overall worse survival rate in patients with lower-risk MDS, but not in higher-risk disease.

The IDH-1 inhibitor ivosidenib is currently FDA- and EMA-approved for patients with relapsed/refractory AML or chemotherapy-ineligible patients with AML with sensitizing IDH-1 mutations. In October of 2023, ivosidenib was approved for relapsed/refractory MDS with a sensitizing IDH1 mutation, based on results from an ongoing phase I trial (NCT02074839). The IDIOME trial is an ongoing phase II trial exploring the safety and efficacy of ivosidenib in participants with higher-risk MDS. The trial encompasses 3 arms: (A) high-risk MDS after azacitidine failure following 6 cycles; (B) higher-risk, treatment-naïve MDS without life-threatening cytopenia; and (C) lower-risk MDS with anemia who did not respond to ESA therapy. The interim results, presented at the American Society of Hematology (ASH)’s annual meeting in 2021, showed an ORR of 54 percent in arm A, 91 percent in arm B and 50 percent in arm C [76]. Unsurprisingly, the treatment-naïve patients had a superior response.

Enasidenib, an inhibitor of IDH-2, is FDA-approved for the treatment of relapsed/refractory AML patients who harbor a sensitizing IDH-2 mutation, with a response rate of nearly 40 percent in this setting [77], although this drug is not currently approved in Canada or Europe. DiNardo et al. investigated the efficacy of enasidenib in MDS harboring an IDH-2 mutation in a phase II trial with 2 arms: enasidenib in combination with azacitidine as an upfront therapy or as a monotherapy in those previously treated with HMA. Encouragingly, the overall response rate was 74 percent in the upfront setting and 35 percent in the previously treated group [78]. Approximately 16 percent experienced differentiation syndrome associated with IDH inhibitor treatment in this study. Larger trials are needed to confirm these results, as well as to determine the appropriate setting for IDH directed therapy in MDS. 

### 6.7. Novel Agents

Despite expanding treatment options, the prognosis of patients with higher-risk MDS upon HMA failure remains poor. Targeted therapies for patients with MDS are continuously being investigated. There have been a number of initially promising biologic targets in the treatment of MDS, albeit few have been approved to date. Candidate drugs have been developed that target a variety of cellular pathways, including the Hedgehog signaling pathway, the RAS pathway, the ubiquitination pathway, the inhibition of phagocytosis (targeting CD47), the process of stem cell renewal, the dysfunction TP53 protein and others.

Glasdegib is an oral inhibitor of the transmembrane G protein-coupled receptor Smoothened, a component of the Hedgehog signaling pathway that functions as an oncogene, ultimately promoting leukemic cell survival. A phase II, single-center open-label trial assessing the impact of glasdegib in participants with MDS or CMML found an overall 6 percent response rate with an acceptable safety profile [79]. A 2019 study by Cortes et al. randomized 132 participants with AML or higher-risk MDS to receive a low dose of cytarabine with or without glasdegib. The median survival time was 8.8 months for those receiving both glasdegib and low-dose cytarabine compared to 4.9 months for those receiving low-dose cytarabine alone. Among those with higher-risk MDS , the combination therapy showed an overall response rate of 20 percent, whereas cytarabine alone showed a response rate of 0 percent [80]. It should be noted that 35 percent of the participants discontinued the study due to adverse effects. Unfortunately, these response rates are not particularly promising, although further clinical studies are needed to define the role of glasdegib in the treatment of higher-risk MDS, including potentially as part of combination therapy.

Rigosertib acts on the RAS signaling pathway by binding to the RAS-binding domain of multiple kinases including RAF, PI3K and PLK. Preclinical trials demonstrated in vitro mitotic arrest and apoptosis in MDS cells. A 2016 randomized, controlled, phase III trial compared rigosertib to best supportive care in participants who failed to respond to, relapsed after or were ineligible for bone marrow transplantation. There was no significant difference in terms of OS between the study arms. A subgroup analysis did demonstrate a possible benefit of rigosertib in participants with very-high-risk disease, participants with monosomy 7 or trisomy 8, and participants with HMA failure [81]. Further investigation into rigosertib may be warranted in the very-high-risk or relapsed setting.

Pevonedistat targets the proteasome pathway by activating NEDD8, a ubiquitin-like protein that targets the cullin subunits of E3 ubiquitin ligases, resulting in the activation of ubiquitination [82]. In 2023, De Carvalho et al. published data suggesting that certain genes involved in the ubiquitination pathway are differentially expressed in patients with MDS. For example, expression of UBE2O, UBE2T and USP7 were increased while USP15 expression was decreased in marrow mononuclear cells [83], suggesting utility in targeting the ubiquitin–proteasome pathway in patients with MDS. In 2021, Sekeres et al. demonstrated that the addition of pevonedistat to azacitidine led to an improvement in OS compared to azacitidine alone (with a median OS of 23.9 months vs. 19.1 months) among participants with higher-risk MDS. Pevonedistat plus azacitidine led to a longer event-free survival (EFS) time compared with azacitidine alone, and the duration of response was also markedly improved in the combination arm with an increased CR rate [84]. The phase III PANTHER study demonstrated no OS benefit with the addition of pevonedistat to azacitidine in the front-line setting in an intent-to-treat population (including participants with low blast AML, MDS and CMML); however, a post hoc analysis demonstrated a modest but statistically significant OS benefit for higher-risk MDS participants after 3 cycles (23.8 vs. 20.6 months) and after 6 cycles (27.1 vs. 22.5 months) cycles [85]. Although larger MDS trials are needed, its efficacy has clearly been limited.

Magrolimab is a first-in-class monoclonal antibody directed against CD47, intended to promote the macrophage-mediated phagocytosis of cancerous cells. A phase Ib study by Sallman et al. explored the addition of magrolimab to HMA in participants with IPSS-R intermediate- to very-high-risk MDS. Magrolimab plus azacitidine resulted in an ORR of 75 percent, with a 33 percent CR rate. Notably, there was a CR of 40 percent among participants with a TP53 mutation. The authors note that over one third of the study participants would go on to have an allo HSCT with a 77 percent OS at 2 years. The drug had a reasonable safety profile, with constipation, thrombocytopenia and anemia being the most commonly reported AEs [86]. The phase III ENHANCE study was a randomized, double-blinded, placebo-controlled trial that included 539 IPSS-R intermediate-, high- or very high-risk treatment-naïve participants, randomized to receive azacitidine with or without magrolimab. Disappointingly, in July of 2023, the trial was discontinued due to futility [87].

Sabatolimab is a monoclonal antibody that targets T-cell immunoglobulin domain and mucin domain-3 (TIM-3), a membrane receptor expressed by myeloid cells and most AML blasts but not by normal hematopoietic stem cells. TIM-3 is believed to play a role in leukemic stem cell self-renewal. It is also expressed on T-cells and dendritic cells and is involved in the downregulation of cell-mediated immunity. As such, this protein represents a rational therapeutic target [88]. In preclinical studies, sabatolimab showed efficacy by mediating immune activation and phagocytosis of myeloblasts in vitro. 2 clinical trials were designed to test the efficacy and safety of front-line treatment with sabatolimab in combination with HMA in participants with IPSS-R intermediate- to very-high-risk MDS: the phase II STIMULUS-MDS1 trial and the phase III STIMULUS-MDS2 trial, which also included participants with CMML [89]. The phase II results were published in February 2024, demonstrating that the addition of Sabatolimab to HMA therapy did not result in an improvement in the CR or PFS [90], and on January 31 Novartis announced the discontinuation of their investigation into sabatolimab. Similar to magrolimab, the lack of efficacy of sabatolimab demonstrates the failure of an exciting biologic mechanism to translate into clinic utility and should remind us of the importance of larger clinical trials.

Eprenetapopt (APR-246) is a small organic molecule thought to bind to the mutant p53 protein and induce a conformational change that results in restored functionality. p53-directed treatment represents an unmet need in the current treatment paradigm given its adverse impact on prognosis. Treatment with eprenetapopt alone results in in-vitro tumor cell death and is believed to act synergistically with HMA in human myeloid cell lines [91], raising hope for the use of p53 activators in MDS. A phase Ib/II clinical trial published by Sallman et al. in 2021 included 55 participants (44 with MDS or MDS/MPN and 11 with AML) with mutant TP53 and evaluated the safety and efficacy of adding eprenetapopt to HMA therapy. This therapeutic combination showed a 73 percent response rate in MDS, with 50 percent of the participants achieving CR and 58 percent achieving a cytogenetic response [92]. Importantly, 35 percent of the participants underwent allo HSCT, with a median overall survival of 14.7 months. It is notable that approximately one-third of the participants in this trial experienced febrile neutropenia, underscoring the challenges of p53 reactivation therapy.

### 6.8. Potential Targetable Pathways: So Many Roads

Although a number of experimental agents have been tested, results in clinical trials have been disappointing. Nonetheless, targeting novel pathways continues to generate excitement. Potential future targets include the exploitation of the apoptotic pathway, targeting inflammatory mediators and immune checkpoint inhibition.

Myeloid cell leukemia-1 (MCL-1) is an anti-apoptotic protein that is a member of the BCL-2 family and is known to be overexpressed in various malignancies. The successful targeting of this pathway has been previously demonstrated with venetoclax in hematologic malignancies such as CLL and AML, and several MCL-1 inhibitors have been tested in the pre-clinical setting and are now entering in early-phase clinical trials for hematologic malignancies, including MDS [93].

Immune checkpoint inhibitors (ICI) interfere with immune downregulation through several mechanisms, including by targeting the CTLA-4 and PD-1 pathway. ICIs have shifted the treatment paradigm in recent years for certain malignancies, such lung cancer. However, they currently have no role in the routine treatment of patients with MDS. Some early-phase clinical trials using pembrolizumab alone after HMA failure [94] or the combination of azacitidine and nivolumab in newly diagnosed patients with MDS [95] have been disappointing. There is an ongoing trial looking at ipilimumab and nivolumab, alone and in combination with azacitidine, including an arm with all 3 drugs (NCT02530463) which is scheduled to be completed in 2025. There are also ongoing trials exploring immunotherapy in addition to other agents, such as in combination with a histone deacetylase inhibitor (HDACi) after HMA failure (NCT02936752). Therefore, we will watch immunotherapy’s progress with interest.

Inflammatory pathway targets may also prove a promising therapy in patients with MDS. Tomaralimab is a monoclonal antibody targeting the innate immune sensor TLR2, which has been shown to be upregulated in blast cells in MDS. In a phase I/II trial including heavily pre-treated (third line or beyond), transfusion-dependent participants with lower-risk MDS, Garcia Manero et al. showed that tomaralimab induced transfusion dependence in half of the participants [96]. There is an ongoing phase I/IIa study evaluating inhibition of IRAK4, a protein kinase involved in innate immunity via TLR signaling, with the oral agent emavusertib with or without venetoclax in participants with higher-risk MDS or AML (NCT0427876800).

## 7. Conclusions

Although significant progress has been made in our understanding of the biology of MDS over recent years, at the present moment allo HSCT remains the only potentially curative therapy. With ongoing research, we anticipate that clinical, laboratory and molecular data will be increasingly utilized in clinical decision making, ultimately leading to more effective and less toxic targeted interventions as we work to decrease morbidity and improve survival for our patients with MDS.

## Figures and Tables

**Table 1:** List of common genetic mutations seen in MDS with approximate frequency and selected characteristics. Asterisk (*) indicates founding lesion per ICC 2022. RS, ring sideroblasts. CK, complex karyotype. AML, acute myeloid leukemia. CMML, chronic myelomonocytic leukemia [2,3,4,5,6,7].

Gene	Pathway/Function	Frequency (Estimated % of MDS Cases)	Comments
Homo sapiens additional sex combs like 1 (ASXL1) *	Chromatin remodeling	15–25%	Less favorable prognosis
BCL6 corepressor (BCOR) *	Negative regulator of transcription	4–6%	Unclear prognostic value
Enhancer of zeste homolog 2 (EZH2) *	Histone methylation	5–10%	Less favorable prognosis
Runt-related transcription factor 1 (RUNX1) *	Transcription factor	10%	Less favorable prognosis
Splicing factor 3B subunit 1 (SF3B1) *	RNA Splicing	20%	Associated with RS phenotype
Serine/arginine-rich splicing factor 2 (SRSF2) *	RNA Splicing	10%	Less favorable prognosis
The stromal antigen 2 (STAG2) *	Multiple mechanisms/part of cohesion complex	8%	Unclear prognostic value
U2 small nuclear RNA auxiliary factor 1 (U2AF1) *	RNA splicing	8–12%	Less favorable prognosis
Zinc finger (CCCH type), RNA-binding motif and serine/arginine rich 2 (ZRSR2) *	RNA splicing	5–10%	Less favorable prognosis
DNA (cytosine-5)-methyltransferase 3A (DNMT3A)	DNA methylation	12–18%	Less favorable prognosis
Tumor protein P53 (TP53)	Cell cycle regulator	5–18%	Less favorable prognosis, associated with CK
Tet methylcytosine dioxygenase 2 (TET2)	DNA methylation	>20%	Often involves insertions/deletion; unclear prognositic value
Isocitrate dehydrogenase 1/2 (IDH1/2)	DNA methylation (via TCA byproduct build-up)	<5%	More commonly seen in AML. Also seen in gliomas. Prognostication yet to be eluciated in MDS
Casitas B-lineage lymphoma (CBL)	E3 ubiquitin ligase	<5%	More frequently seen in CMML and juvenile myelomonocytic leukemia, unclear prognostic value
Cut like homeobox 1 (CUX1)	Transcription regulation	up to 15%	Deletions seen in monosomy 7 and del7q due to location on long arm of Ch7
Janus kinase 2 (JAK2)	Cytokine receptor signaling	<5%	Nonreceptor tyrosine kinase, unclear prognostic value
Translocation-Ets-leukemia virus (ETV6)	Transcription regulation	<5%	Less favorable prognosis
Neurofibromatosis 1 (NF1)	RAS signaling pathway	<5%	Estimated to be mutated in 1.6% MDS cases
Neuroblastoma RAS viral oncogene homolog (NRAS)	RAS signaling pathway	5-10%	More frequently seen in CMML and juvenile myelomonocytic leukemia, less favorable prognosis

**Table 2:** MDS subtypes within the WHO5 and ICC 2022 classification systems. Both groups recognize presence of del(5q) and SF3B1 mutation; however WHO5 also recognizes the presence of bi-allelic inactivation of TP53 as a distinct subcategory. Both systems take into account the presence of increased blasts. The MDS/AML category within the ICC 2022 schema includes presence of 10 percent or more blasts without an AML defining molecular lesion [9,18].

WHO 5th Edition (WHO5)	ICC 2022
MDS with defining genetic abnormalities	MDS with mutated *SF3B1* (MDS-*SF3B1*)
MDS with low blasts and *SF3B1* mutation (MDS-*SF3B1*)	
MDS with low blasts and isolated 5q deletion (MDS-5q)	MDS with del(5q) [MDS-del(5q)]
MDS with biallelic *TP53* inactivation (MDS-bi*TP53*)	
	MDS, NOS without dysplasia
MDS, morphologically defined	MDS, NOS with single lineage dysplasia
MDS with low blasts (MDS-LB)	
MDS, hypoplastic (MDS-h)	MDS, NOS, with multilineage dysplasia
MDS with increased blasts (MDS-IB)	
MDS-IB1 (5% to 9% blasts in marrow or peripheral blood)	MDS with excess blasts (MDS-EB) (5–9% blasts in marrow or 2–9% in peripheral blood)
MDS-IB2 (10 to 19% blasts in marrow or peripheral blood)	
MDS with fibrosis (MDS-f)	MDS/AML (10 to 19% blasts or more in marrow or peripheral blood without an AML defining lesion).

## Data Availability

No new data were created or analyzed in this study. Data sharing is not applicable to this article.
